# Key concepts in biosimilar medicines: **What physicians must know**

**DOI:** 10.14744/nci.2021.84669

**Published:** 2022-02-10

**Authors:** Alper B. Iskit

**Affiliations:** Department of Pharmacology, Hacettepe University Faculty of Medicine, Ankara, Turkey

**Keywords:** Biologic drugs, biosimilar, biotechnology

## Abstract

Biologics’ are a class of medications produced by living cells using recombinant DNA technology. Biologics have had an important impact in many areas of medicine, and in particular in rheumatology and oncology. However, the high cost of these agents is a growing concern, particularly as more products become available and their use for the treatment of immune-mediated inflammatory diseases continues to expand. Biosimilars, also called follow-on biologics, have been viewed as a potential cost-saving alternative to traditional therapies. Currently, a product can be considered biosimilar to a reference product if there are no clinically meaningful differences in terms of safety, purity, and potency. In this review, the most important key concepts about biosimilars were summarized for physicians emphasizing the status in Turkey.

**Figure 1.**
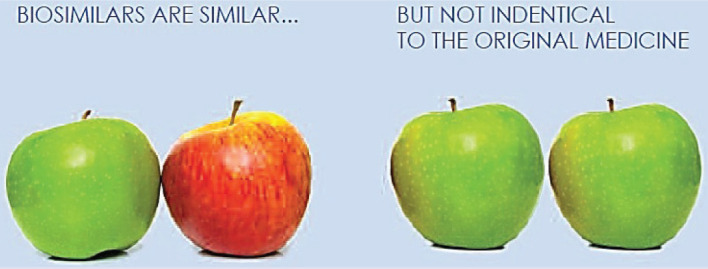
Biosimilars are not identical to the original drug.

**Highlight key points**

• Biosimilars are not the same as generics.
• Biosimilars that have been approved have been proven to be safe and effective.
• Biosimilars’ economics are undeniable.
• Biosimilars can lead to innovation among reference product manufacturers.

**Figure 2.**
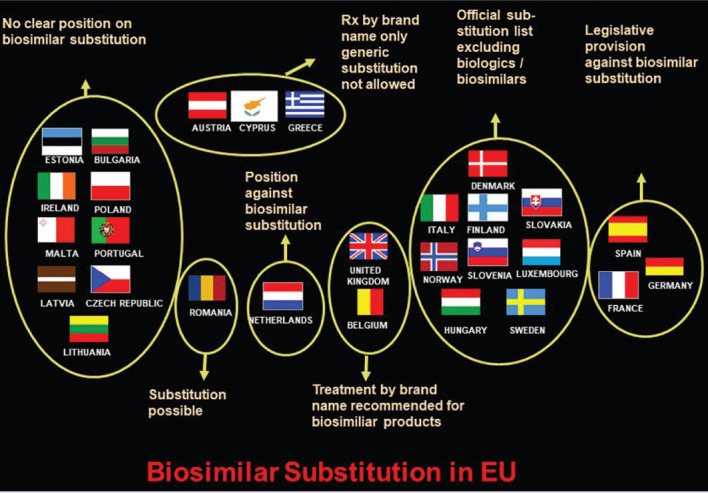
European Medicines Agency communicated its reservations regarding interchangeability for European Union Member States but the final decision was left to national authorities

**A**lthough biological medicinal products have fundamentally changed the therapy of patients who have life-threatening and debilitating diseases, the discovery potential of new biological products for unmet needs is still high. Biotechnological methods enable the development of novel treatments for many highly serious diseases, clinically and epidemiologically, and make it possible to create solutions for the growing healthcare needs of the population. The production of biotechnological medicines, which began with insulin in 1982, continued with growth hormones, erythropoietin, various interferons, enzymes, coagulation factors, vaccines, and monoclonal antibodies (trastuzumab, abatacept, adalimumab, etanercept, infliximab, etc.) which carry great importance for healthcare expenditures.

Unlike conventional medicines, biotechnological medicines are products produced from organisms and live systems through biotechnological processes such as recombinant DNA technology or monoclonal antibody methods instead of chemical compounds. These products are larger and more complex compared to products manufactured by chemical synthesis and obtained using living systems, usually by reproducing a protein inside a living cell (e.g. a bacteria or mammal cell). The attributes of biotechnological medicines are largely dependent on the conditions of the manufacturing process, and as they say “the process is the product.” Even minor changes in the manufacturing process may alter the final product, requiring, therefore, the manufacturing processes of biotherapeutic products to be well designed, robust, reliable, and fully controlled. Good manufacturing practice, validation, and predefined specifications are essential for ensuring the safety and effectiveness of these products [1].

Biosimilars are similar versions produced following the patent expiry of original biotechnological products (formulation). Biosimilar medicines are similar to but not identical and the same as original medicines in terms of biological medicines (Fig. 1).

## Biosimilars are not Generics

A biosimilar is a biological product that has been developed to be substantially similar to an existing biological medicinal product (the original, “reference” product). Biosimilars are also biotechnological products produced from living organisms such as the reference product. Their molecule structure is also huge and complicated. Because each biotechnological product has a unique manufacturing process, it is impossible to manufacture an identical copy of biological medicine. The biosimilars may therefore be only “similar” to the reference product, and it is recognized that specific differences may exist between the two products, due to process differences. Because they are manufactured using different cell lines and manufacturing processes, a biosimilar cannot be identical to its reference product, but the two are similar in terms of quality, safety, and efficacy. Furthermore, it is also determined that some biosimilar products do not cause the same side effects as references. Variations that may occur in the three-dimensional structure during production, in the amount of acid-base variants, and the glycosylation profile (post-translational modifications) are regarded as minor changes, but they may lead to serious and significant differences in the effect (and side effects) of the end-product which may be the reason of fatal allergic reactions (immunogenicity) [2].

Generic (equivalent) medicines are small molecules with a chemically fixed structure that are produced via chemical synthesis and are completely the same as the reference drug. Therefore, biosimilars cannot be regarded as generic products (copies of chemical medicines).

## Biosimilars Are Essential

The major load brought by biotechnological medicines on the economy canalizes healthcare authorities towards biosimilar medicines. The first five biosimilar medicines were registered in 2006 and 2007 following the patent expiry of the original medicines in Europe. The absence of long-term safety data for biosimilars obliged healthcare authorities to issue certain arrangements regarding such types of medicines. The European Medicines Agency (EMA) made the relevant legal arrangements via publication of product-specific guidelines and consequently began to issue the first registrations [3, 4]. With the development of biosimilar medicines, it is aimed to meet the high need for biological medicines across the world at a lower cost, ensure competition and incentivize research and development. The fact that this is a global matter has been confirmed with the World Health Organization (WHO’s) general principles are and the guidelines issued in 2016 on the approach regarding biosimilars of monoclonal antibodies [5].

## Registration Of Biosimilars

To produce biosimilar medicines, it is necessary to carry out all relevant trials including the clinical trials of that product. It should be proven that these products display similar characteristics in terms of quality, safety, and efficacy with the reference product. Physicochemical and biological comparisons (quality of medicine), comparative non-clinical comparisons (*in vitro*, preclinical, toxicity, etc.), and clinical comparison (human impact) studies are conducted for this purpose. In 2005, EMA launched a new procedure for the first time specifically for the registration of biosimilar medicines “EMA guideline on biosimilar medicinal products” [6, 7]. Further, in 2009, the WHO developed guidelines for the development and evaluation of biosimilar medicines “WHO guideline on the evaluation of similar biotherapeutic products.” The minimum standards outlined in the WHO guidelines have been developed to both provide a shorter process for registration and preserve quality, safety [5].

Science-based registration standards for medicines are crucial for patient safety and biosimilar medicines must have different registration standards than generics, owing to the complex structures of biological medicines. The evaluation of biosimilars requires a way of registration that is defined for specific characteristics of biological medicinal products and manufacturing processes. Biosimilars should be analytically compared with the reference product to identify and justify the differences, and the product’s safety and efficacy must be supported with clinical trials. EMA, Food and Drug Administration, and especially the other pharmaceutical authorities’ approach toward the standards and requirements of clinical trials is not homogenous and is the main reason for ongoing discussions across the world in relation to biosimilars, especially on efficacy and safety [8]. This situation reveals another important concept called “similar biotherapeutic products” (SBPs) which is used for products licensed under conditions that did not meet international regulatory expectations in some countries. A serious problem has been identified in some countries where biological products have been licensed on data that did not meet international regulatory expectations. Sometimes, these copy products have even been licensed as simple generics. Nomenclature for these products is confusing and traceability is poor. Coexistence on the market at the same time of inappropriately licensed copy products and true biosimilars is a matter of concern. It should be noted that “non-originator biologicals” approved in Russia and Turkey, “similar biologics” approved in India, “biocomparables” approved in Mexico, “SBPs” approved in Latin America, “biogenerics” approved in Iran, copy biologicals approved in China and “medicamento biológico similares” approved in Argentina might not have been authorized if they had been subjected to the strict regulatory processes required for approval of biosimilars in the European Union (EU) and the USA.

## Interchangeability and Substitution in Biosimilars

Interchangeability is a term indicating that a medicine may be replaced with another medicine proven to be equivalent. To substitute a product with another product at pharmacies, it should be demonstrated that those products are interchangeable and the healthcare authority should adopt a decision in this direction [9].

In principle, it is globally accepted that generic products are interchangeable with original products. However, this is debatable especially for physicians in biosimilar products. EMA stated its reservations regarding interchangeability for the EU countries, but the final decision was left to the national authorities of member countries within time. Therefore, there are different approaches regarding the substitution of biosimilars instead of the original biological medicine at pharmacies (Fig. 2).

### Countries where the Substitution of Biosimilars at Pharmacies is Legally Prevented

In 2011, AFSSAPFS in France (French National Medicines Agency) officially declared that biosimilars of biological/biotechnological products may not be regarded as generics and that original products and biosimilars should not be substituted at the pharmacy level.

The substitution of biological medicines at pharmacies in Germany is regarded as suitable only if the product is produced from the same source with the same production technique and displays no difference at all. This applies only for co-marketed products produced by the same company in the same manufacturing site but is marketed by different companies and products produced by different companies with different production techniques cannot be substituted at pharmacies. No substitution is allowed in Spain.

### Countries with an Official List of Substitutable Medicines but in which Biosimilar Products are Excluded from this List

The products that may be substituted at pharmacies are clarified by a list (automatic Substitution List) published by AIFD (Italian National Medicines Agency) in Italy. There are no biosimilar products on the referred list and AIFA declared that biological products should not be substituted at pharmacies due to safety reasons.

There is a similar implementation also in Denmark, Norway, Finland, Sweden, Slovenia, Hungary, Slovakia, and Luxembourg.

### Countries where Prescribing is made by Product Name and the Substitution of Biosimilars at Pharmacy Level is not Possible

Products are prescribed with the active substance in the UK and maybe interchanged with other products with the same active substance at the pharmacy level. British Medicines and Healthcare Products Regulatory Agency requires biological products to be prescribed by brand name instead of the active substance and thus biological medicines cannot be substituted at pharmacies.

Biosimilar products are not legally allowed to be substituted at pharmacies in Greece.

There is a similar implementation also in Belgium and Austria.

### Countries where the Decision to Substitute or not Biological/Biosimilar Products at Pharmacy Level is Left to Physicians

Portugal and the Netherlands.

### Countries where there is Official Documentation or no Clear Position on the Substitution of Biosimilars at Pharmacies

Estonia, Portugal, Ireland, Lithuania, Bulgaria, Czech Republic, Malta allow substitution at pharmacies.

In the US, the following is required to enable a biosimilar to be interchanged with a reference product:

A biosimilar product administered to any patient should yield the same clinical results as the reference product and should not pose a risk higher than the risk caused by the reference product in terms of safety. Furthermore, it is requested that there should be no extra risk posed by the transition from the original medicine to a biosimilar medicine. A biosimilar product observed to fulfill these criteria and defined as interchangeable may be substituted with the reference product without the approval of the physician who has prescribed the reference medicine.

Currently, there is no arrangement in our country indicating that similar products cannot be dispensed in place of the reference product. In other words, the reference product and the biosimilar product are regarded as interchangeable. However, most physicians defend the “right for freedom of treatment” worldwide and would like to avail of the flexibility to request the biological product they have selected upon deciding on the medicine with which they will continue with the treatment. The most important justification is that biosimilars are not generics are specified at the beginning of this article.

## Product Information and Safety Issues in Biosimilars (Risk Management Plan and Pharmacovigilance)

Biosimilar products should be placed on the market after the expiration of patent and data protection periods of the reference biologic products. An environment providing patent protection and appropriate data exclusivity will facilitate the development of innovative products ensuring the new treatments for patients and new reference products for new biosimilars.

The data obtained from the studies of the biosimilar product itself should be provided comparatively with the data of the original product in the informative guidelines of biosimilar products (SPCs/PIL).

A “risk management plan” should always be drawn up for a biosimilar product. A “risk management plan” is a key instrument for identifying and tracking the potential risks arising from the experience with a limited number of newly approved medicinal products. A risk management plan including post-marketing pharmacovigilance activities and immunogenicity studies is necessary for enabling a proper evaluation of the risk/benefit profile of a biosimilar product and is mandatory for risk minimization [9].

Pharmacovigilance aims to identify, evaluate and prevent undesired effects of a product on the market. To conduct accurate pharmacovigilance, it is important for physicians, pharmacists, and patients to easily distinguish original and biosimilar products. For accurate pharmacovigilance, it is important for physicians, pharmacists, and patients to be able to conveniently identify a biopharmaceutical product. If the same International Nonproprietary Name is used for multiple products, it will be impossible to properly associate adverse events with a specific product. To ensure definitive identification of a suspect product, biological products should be identifiable on the Adverse Event Report, with the brand. In 2016, EMA published a pharmacovigilance guideline specifically for biological medicines and underlined the importance of this topic [10].

## Extrapolation of Indications in Biosimilars

If their mechanisms of action provided for treatment is common, biotechnological products may be used in multiple indications. If the medicine is proven to be clinically similar to the reference product in its main indication may enable the expansion of safety and efficacy data upon comprising also the secondary indications approved by the medicine. This is called extrapolation. If the medicine will be used in different systems for the treatment of different diseases, the only clinical trial conducted for the main indication may lead to debates in terms of the utilization of the secondary indications of the medicine, especially among clinician medical doctors from different disciplines. Therefore, the scientific justification of the extrapolation should always be substantiated with preclinical studies or other tests.

## Registration Requirements and Conditions of Biosimilars in Turkey

It is estimated that the biotechnological medicines market will grow by 12–15% annually worldwide in the upcoming years. The “guideline on biosimilar medicinal products” published in Turkey in 2008 has designated also the registration criteria of biosimilar medicines (Table 1). Increasing the number of biosimilar medicines will reduce treatment costs and raise the accessibility of patients to medicines. However, especially clinicians should be careful regarding the quality, safety, and efficacy of biosimilar medicines [11]. Turkey has not any official position paper on interchangeability, switching, and substitution.

**Table 1.** Comparison of the registration application for generic products, biosimilar products, and new (original) products

	Classical equivalent product	Biosimilar product	New product (Complete dossier)
Quality	Comparison of the “Dossier information of Complete and Independent Product”	“Dossier information of Complete and Independent Product” Extensive comparison with the reference product	Dossier information of Complete and independent product
Preclinical	-	Abridged program, subchronic toxicity study affiliated with the complexity of the molecule (4 weeks). Local tolerance, PK/PD (pharmacokinetic/pharmacodynamic) study	Preclinical complete study
Clinical	Bioequivalence study	Phase I: PK/PD (pharmacokinetic/pharmacodynamic) study Phase II study is not required. Where necessary, phase III study for each indication Risk management plan	Phase I Phase II Phase III studies in all indications Risk management plan

To enable the production of biological medicines which constitute a major part of pharmaceutical expenditures in Turkey and the share of which is expected to grow exponentially in upcoming years, the Scientific and Technological Research Council of Turkey (TUBITAK) accepted project applications for local development and production of biosimilar medicines within the scope of the Support Program for Public Research Projects (1007 program). Moreover, the amendments made in the pricing notification for incentivizing the production of biosimilar products in our country enable biosimilar products, even if they are imported, to be priced as 100% the same as the original product [12].

Following the publication of the “Guideline on Biosimilar Medicinal Products,” dated August 2008, the first biosimilars began to be introduced in Turkey. Some products are directly imported (as finished products) while others are imported as raw materials and filled in Turkey [12]. The guideline has also been improved and updated recently in 2021 [13].

## Awareness of the Therapeutic Aspects of Biosimilars from a Clinical Perspective

First of all, all healthcare professionals in our country should be aware of biosimilars and should never regard these medicines as generics. The generic medicine approach may be the reason and explanation of different negative or positive pharmacological responses obtained with biosimilars (lack of efficacy, unexpected side effects, toxicity, excessive effect, etc.) [14, 15].
